# Patient care, integration and collaboration of physician associates in multiprofessional teams: A mixed methods study

**DOI:** 10.1002/nop2.1655

**Published:** 2023-02-20

**Authors:** Stephanie Howard Wilsher, Audrey Gibbs, Joanne Reed, Rebecca Baker, Susanne Lindqvist

**Affiliations:** ^1^ Centre for Interprofessional Practice University of East Anglia Norwich UK; ^2^ Norwich Medical School University of East Anglia Norwich UK; ^3^ James Paget University Hospital Gorleston UK; ^4^ Norfolk and Norwich University Hospital Norwich UK

**Keywords:** clinical effectiveness, health service research, interprofessional education, multiprofessional practice, patient perspectives

## Abstract

**Aims:**

The aim of the study was to explore the physician associate role in patient care, integration and collaboration with team members, within the hospital setting.

**Design:**

Convergent mixed methods case study design.

**Methods:**

Questionnaires with some open‐ended questions and semi‐structured interviews were analysed with descriptive statistics and thematic analysis.

**Results:**

Participants included 12 physician associates, 31 health professionals and 14 patients/relatives. Physician associates provide effective, safe and, importantly, continuity of care and patients received patient‐centred care. Integration into teams was variable, and there was a lack of knowledge about the physician associate role amongst staff and patients. Views towards physician associates were mostly positive, but support for physician associates differed across the three hospitals.

**Conclusion:**

This study further consolidates the role of physician associates to multiprofessional teams and patient care and emphasises the importance of providing support to individuals and teams when integrating new professions. Interprofessional learning throughout healthcare careers can develop interprofessional working within multiprofessional teams.

**Impact:**

Leaders in healthcare will see that clarity about the role of physician associates must be given to staff members and patients. Employers and team members will see the need to properly integrate new professions and team members within the workplace and to enhance professional identities. The research will also impact on educational establishments to provide more interprofessional training.

**Patient and Public Involvement:**

There is no patient and public involvement.

## 1 INTRODUCTION

The global shortage of medical and nursing workforce lead to the development of several new roles, for example nursing associates, advanced nurse practitioners and physician associates (PAs)—a strategic move to solve an ever‐increasing challenge and care need (WHO, 2016). The PA role is generally accepted by other healthcare professionals (Halter et al., 2018), and their contributions to multiprofessional teams are recognised. Patients are mainly satisfied with the care they receive from PAs (Hooker et al., 2019). PAs are viewed as approachable, informative and patients trust them (Drennan et al., 2019). However, confusion about the PA role is prevalent amongst staff and patients, which can lead to patient dissatisfaction (Drennan et al., 2019; Halter et al., 2018; Taylor et al., 2019) and poor integration into MDTs (Roberts et al., 2019).

In a recent survey of qualified and student PAs, although 80% were satisfied and enjoyed their work, almost 20% said they were despondent in their role and 30% felt excessive work pressure (Ritsema, 2018). It may be lack of clarity around roles that is challenging for new professions (Roberts et al., 2019).

### 1.1 Background

PAs were first introduced in North America in the 1960 s to address medical shortages in primary care (Mittman et al., 2002). Since 2018, other countries have incorporated the PA role into their healthcare systems where they work in a variety of clinical contexts (Rick & Ballweg, 2017). In the Netherlands, research looking into the PA role have highlighted the need to understand how this new profession compares to and complements medical doctors to ensure optimal care delivery (Timmermans et al., 2016). PAs have been practising in the United Kingdom (UK) for 10 years, but only since 2018 in the region, which was the focus of this study.

In 2020, over 1550 PAs were on the Physician Associate Managed Voluntary Register (PAMVR) to work in England, and over 130 worked in the region of this study. At present, PAs are not formally regulated in England, but are required to complete 50 hours of continuing professional development (CPD) to remain on the PAMVR (Faculty of Physician Associates, 2021). Lack of regulation and governance, together with absence of clarity around their role and responsibilities, are common reasons for some NHS hospitals not fully embracing this new profession in their workforce (Drennan et al., 2019).

Drennan et al. (2019) conducted a comprehensive study of PAs in six hospitals across three regions in England. Most of these hospitals were urbanised areas around large cities. Hence, there are still many locations where PAs are only recently being employed and where the understanding of PAs contribution to collaborative care in hospitals remains unclear. Furthermore, there is little empirical findings available that tell us about their integration into existing teams.

Here, we extend the current evidence of PAs contribution to care within the hospital setting, their integration into teams and their collaboration with team members and patients. Our study focused on hospitals in a rural location, where PAs have been recently employed, thus providing further understanding of introduction of PAs into acute hospital settings.

## 2 THE STUDY

### 2.1 Aim

To explore the PA role in the care of patients, their integration into teams, and their collaboration with team members and patients, within the hospital setting.

### 2.2 Research team and reflexivity

The lead author (SHW) and SL were the researchers with most input into the study. Both researchers are female and hold PhDs. SL is a world advocate for interprofessional development and training, with over 30 years' experience. SHW has over 10 years in qualitative research and had no knowledge of the PA role prior to the research, therefore was cognisant to potential bias of doctor versus PA in the workforce. One team member (AG) is director of PA training, another member (JR) is a PA, and RB is an experienced researcher who began the study.

A rapport was developed with participants before interview, when the lead researcher (SHW) explained the reasons for the research, their interest and possible assumptions about the PA role in health care.

### 2.3 Study design

A mixed method convergent parallel (Creswell, 2011) case study design (Yin, 2009) was chosen. The study was informed by a pragmatic realist approach to understand how people perform within their work system (Robson, 2002).

#### 2.3.1 Setting

Three NHS acute hospitals in a rural area of the UK where PAs have been employed since 2018. The hospitals have a bed capacity ranging between 500 and 1200 and employ between 3000 and 6000 staff members.

#### 2.3.2 Participant selection

All PAs (20) working in the three hospitals at the start of this study were invited by email to take part. Staff who were working with them as part of the multiprofessional team also received an email invitation. Twenty‐five patients, or relatives of patients who had been treated by each PA in the three hospitals, were approached by senior ward staff and over a one‐week period.

#### 2.3.3 Data collection

The lead author (SHW) visited senior clinical collaborators in each hospital to ask them to recruit PAs, staff working with PAs and patients treated by PAs. All data were collected in 2020.

#### 2.3.4 Questionnaires

Questionnaires were designed on topics for each participant group (Table 1), included open‐ended questions and an invitation to take part in the interview.

**TABLE 1:** Questionnaire and interview topics for each participant groups

Participant group	Topics included in the questionnaire
Physician Associate ‐ questionnaire	Perceptions of patient views on the role How they perceived their role in MDT How prepared they felt for the role Perceived their input in the MDT Supervision and CPD training Satisfaction about their role
Extra topics for interview	How PAs address patients Patient response to PA care Team integration Employment of PAs—barriers/benefits Social acceptance Future of PAs
Staff in MDT's ‐ questionnaire	Patient awareness of PAs Confidence in healthcare provided by PAs PA contribution to the MDT Supervision and CPD training of PAs
Extra topics for interview	Views on PAs employment in NHS Employment of PAs—barriers/benefits Social acceptance Future of PAs
Patients ‐ questionnaire	Knowledge of PAs involved in their care Person‐centredness of their care: approachability, listening skills, explanation and discussion of treatment Perceived safety, both now and future care by a PA
Extra topics for interview	Care received from PAs Team integration

#### 2.3.5 Interviews

Participants who agreed to be interviewed (PAs = 6, staff = 15, patients/relatives = 6) were purposively selected to include diversity within the sample. Volunteers who were not selected received a letter to inform and thank them for their interest in the study. All interviewees gave informed consent prior to interview.

Semi‐structured interviews were designed for each participant group, (Table 1).

Each interview was conducted by telephone, lasted between 15 and 45 minutes and were captured on a digital recorder. Interviews were transcribed verbatim, anonymised and returned to participants for member checking. Two staff and one PA made minor changes to their transcripts. Patients who were interviewed received £20 as ‘thank you’.

### 2.4 Ethical considerations

Ethical approval was gained from the NHS, using the Integrated Research. Application System, application number REDACTED. Approval was granted on condition that a senior clinical collaborator in each hospital would recruit participants.

### 2.5 Data analysis

Closed questions in the questionnaire were aggregated for descriptive analyses of each participant group. Open‐ended questions and interviews were thematically and iteratively analysed following the steps outlined by Braun and Clarke (2006). Preliminary themes were identified across the PAs, staff and patient data sets to address the research questions. Case study analyses were conducted to examine any differences in PA treatment and employment at the three hospitals.

### 2.6 Validity and reliability

Questionnaires and interview topics were drawn from previous research, and formal pre‐study discussions with clinicians, trainers, and a PA. The survey was piloted on six university staff who were familiar with PA practice. Interview transcripts were returned to participants for them to conduct a validity check on the content. Qualitative data were analysed by SHW and independently checked by SL. Two PAs, who were not interviewed, discussed, and helped to refine the preliminary themes with the researchers. In addition, case study design allowed comparison across cases (Yin, 2009).

## 3 FINDINGS

Twelve PAs, 31 staff (clinicians), and 14 patients and relatives returned questionnaires. PAs worked at hospital 1 (*n* = 2), hospital 2 (*n* = 3) and hospital 3 (*n* = 7) and represented different specialities (Table 2).

**TABLE 2:** Departments where physician associates worked

Department	Participants
Acute Medical Unit	4
Surgery	1
Cardiology	2
Geriatrics	1
Accident and Emergency	2
Gastroenterology	1
Ward/Respiratory	1

*Note*: Participants are not segregated by hospital to protect their anonymity.

Clinicians from hospital 1 (*n* = 18), hospital 2 (*n* = 9) and hospital 3 (*n* = 4), ranged from nurse (band 5) roles, junior (foundation year doctor) to senior (consultant).

Thirteen patients and one relative (hospitals 1 and 2) returned questionnaires. Five patients and one carer were female, three patients were male, and five did not report their gender.

Questionnaire responses for PAs, clinicians and patients are presented in Tables 3, 4, 5.

**TABLE 3:** Physician associate responses to statements about their role

Statement	Strongly agree	Agree	Neutral	Disagree	Strongly disagree
I gain job satisfaction from my PA role	3	9	0	0	0
I believe my pre‐registration training prepared me to carry out my role safely	1	10	0	0	1
The supervision I receive enables me to carry out my role safely	0	8	2	0	2
I can attend enough CPD training to maintain my registration	3	5	1	3	0
I have time to discuss the health condition and treatment with the patient	4	6	2	0	0
Patients are aware of the PA role	0	2	1	7	2
Patients realise the difference between PA and doctor	0	1	2	7	2
Patients have confidence in my care	5	7	0	0	0
Patients are satisfied with the care I give them	2	7	0	0	3
I think the team trust me to work independently as appropriate	6	6	0	0	0
My role as PA has made a valuable contribution to the healthcare team	0	8	0	0	4
I am valued by my colleagues on the healthcare team	7	5	0	0	0
I help the healthcare team to work efficiently	6	4	0	0	2
I help the healthcare team to work effectively	7	5	0	0	0

**TABLE 4:** Staff responses to statements about the PA role

Statement	Strongly agree	Agree	Neutral	Disagree	Strongly disagree
The PAs have adequate supervision	6	14	5	5	1
The PAs have sufficient opportunity to attend CPD events	0	14	13	4	0
PAs have time to discuss the health condition and treatment with patients	2	22	6	1	0
Patients are aware of the PA role	0	2	4	20	5
Patients realise the difference between PA and doctor	0	4	6	17	4
Patients have confidence in a PA's care in the absence of a doctor	2	13	11	4	1
PAs provide safe medical care to patients	11	12	4	3	1
The team trust PAs to work independently as appropriate	9	13	3	4	2
PAs make a valuable contribution to the healthcare team	15	9	4	2	1
Having a PA on the team is helpful	19	7	3	2	0
PAs help the healthcare team work efficiently	17	9	3	2	0
PAs help the healthcare team work effectively	18	7	3	3	0
Having a PA on the team promotes good teamwork	12	7	7	2	3

**TABLE 5:** Patient responses to statements about the PA role

Statement	Yes	No	Not reported
Did you know a PA was involved in your care	5	8	1
Did you know about the PA role before your stay in hospital?	1	13	
Do you know more about the PA role following your stay in hospital?	8	6	

Statement	Strongly agree	Agree	Neutral	Disagree	Strongly disagree
The PA was approachable	13		1	0	0
The PA listened to my concerns	13	1	0	0	0
The PA explained things in a way I could understand	12	2	0	0	0
I had time to discuss my health condition and treatment with the PA	10	3	1	0	0
I had confidence in the PA to provide me with safe medical care	11	2	0	0	0
I would be happy for a PA to be involved in my care again in the future	13	1	0	0	0

Four PAs, seven clinicians, four patients and one relative across the three hospitals were interviewed. Synthesised findings are presented using our research enquiries as headings.

### 3.1 The perceived contribution of PAs to patient care

Findings from both questionnaire and interviews showed that PAs provided patient‐centred care and their interactions with patients were valued. PAs were seen to make positive contributions to the patient experience and were mostly thought to give safe patient care, but some staff were concerned about the clinical supervision of PAs.

Patients agreed or strongly agreed that they had confidence in PAs providing safe care, and that they would be happy to be treated by PAs in the future (Table 5). Patients, particularly in hospitals 2 and 3, welcomed the person‐centred care of PAs, who explained everything clearly in layman terms and supported them during their healthcare journey.

Sort of put you at ease, explained everything even to the extent of you know ‘we need to get you scanned’ and [the PA] walked us round to where the scan was going to take place and explained everything and sort of collected us from you know different places and that. So, I thought [the PA] was brilliant. [Patient ID:4, hospital 2]

PAs are trained in the medical model. However, they tend to have close interactions with patients, which participating PAs in this study agreed is important and something that had attracted them to the role.

I feel like you are more there for the patients, rather than just focussing on the medical side of things. [PA ID:1, hospital 3]

All participant groups felt that patients have a better experience in hospital because the PAs spend more time with patients and listen to them. Overall, PAs enhance the patient journey by conducting, and/or organising clinical investigations quickly and efficiently. A key benefit highlighted was the consistent presence of PAs on the wards since this enabled them to get to know patients and vice versa. This was especially important when patients had frequent hospital visits as PAs got to know the patients and could therefore provide continuity of care.

Patients really appreciate it when they see the same face every day, especially when the rest of the medical team could be changing. I have found that the patients will let me know of new symptoms, or how they are feeling that day, because you build up trust over time. [PA ID:2, hospital 2]

On contrary to the positive experiences reported, only 48% surveyed staff felt that patients had confidence in the care provided to them by PAs (Table 4). Despite 74% of staff reporting that PAs provided safe medical care, only 64% agreed or strongly agreed that PAs were adequately supervised in their clinical work (Table 4).

All PAs felt they had appropriate clinical supervision for them to work safely though they felt less supported during night shifts because of work demands, working across departments, fewer staff and new doctors on shift.

Despite the concerns, PAs and consultants had identified departments and roles where they could work to improve patient care. PAs termed this as ‘forging a role’, due to the lack of professional structure for PAs within the interprofessional team. One example of this, was the development of a new role that co‐ordinated discharge of mothers and babies (hospital 1).

### 3.2 The perceived role of PAs within the multiprofessional team

The PA role was seen as varied, and findings demonstrate that they conduct numerous day‐to‐day procedures. The strengths of having PAs as part of the team included the continuity of care, however, some working practices resulted in a ‘trade‐off’ with flexibility.

Interprofessional practice was evident, and PAs had enhanced the team's knowledge. The PAs provided informal training to other clinicians working as part of the team, which offered additional support and stability. However, the introduction of the new PA role had also brought a lack of clarity, with patients not knowing if they were treated by a PA or a doctor. Also, it caused some confusion amongst existing roles. This became particularly evident as duties and training opportunities overlapped with other clinicians.

Staff and PAs identified that the PA role is varied and that it overlaps with nurse and doctor roles. In addition, they agreed that PAs often work across several teams to meet the needs of a patient.

Procedures such as taking blood, doing cannulas, but then slightly more advanced ones as well…also liaising with different teams. A patient never comes in with just one problem…so have a lot of times liaising with other specialities [e.g. Respiratory or Gastroenterology] to make sure that all the different aspects of the patients' care are being met. [PA ID:1, hospital 2]

Most PAs (92%; Table 3) and staff (80%; Table 4) felt that patients would not know what a PA is and how their scope of practice differed from doctors. Indeed, this perception was confirmed by this patient.

Well yes, which took me by surprise because like I say how professional they were, the things they were doing, I assumed they were doctors. [ID:3, hospital 2]

Questionnaire data showed that patients' perceived knowledge about the PA role had increased from one knowing about the PA role when they arrived in hospital to eight knowing about the PA role at the end of their stay (Table 5). This was largely due to PAs introducing themselves to patients and informing them about their treatment, however, patients rarely queried the PAs about their role in patient care.

so I asked [PA] about it and [PA] said that they were not able to write prescriptions themselves, I thought well they were doing everything else and surely, they should be able to prescribe. [Patient ID:3, hospital 2]

Staff (71%) felt PAs could be trusted to work independently and 61% felt that the PAs had promoted the teamwork within their team (Table 4). Eight comments on the survey stated that PAs were helpful and contributed to teamwork. Also, by working core hours, PAs provided departmental familiarity that helped other clinicians, in addition to their contribution to patient care, through being a continuous presence on the wards.

there was a Physician Associate who was there from Monday to Friday nine to 5 pm and yes it was, I think it was a good example of that position being very well utilised, and s/he was kind of constant on the Ward, incredibly useful doing jobs, working very well with the MDT [multidisciplinary team]. [Foundation doctor ID:1, hospital 2]

Most staff and PAs felt that PAs helped with the efficiency (84%) and effectiveness (81%) of the team (Tables 3 and 4). It was also perceived that PAs offered valuable knowledge and support to colleagues, especially to newly qualified doctors.

They [PAs] are a different role to the Foundation years [junior doctors], and I see their real strength as being the stability in the team so they are the people who know however a thing runs and can hand on that knowledge to new doctors who change very often. [Consultant ID:2, hospital 2]

In addition, PAs were also seen to improve multiprofessional practice by learning clinical procedures that are regularly needed in a department that they could manage independently. This was something some staff viewed positively.

[PAs] have independent flexible cystoscopy lists, which would have been something that previously my grade would have done, but it is easier to train the PAs once and they stay for much longer than the length of the surgical trainee rotation. [Mid‐grade doctor ID:3, hospital 1]

PAs felt that as generalist clinicians, they could learn different skills and therefore contribute to various departments, but the downside of this flexibility would be loss of continuity of care, as highlighted by one PA.

I can go where I am needed, but at the same time it's a bit difficult for continuity both for myself and also for the patients as well. [PA ID:1, hospital 2]

Apart from not being regulated, some staff were concerned about less distinction of clinical roles if PAs were given more powers and whether all PAs would fit into the multiprofessional team.

### 3.3 The perceived integration of PAs into existing teams and hospitals

Overall, perceptions of integration and attitudes were good, but there were several variations between hospital 1 compared with hospitals 2 and 3. Comparing the analyses of the three hospitals revealed notable differences in the attitudes towards employment, training and appraisal of PAs.

The lack of clarity of the PA role and how it fits into the wider multiprofessional team appeared to be due to little guidance being provided to staff and patients before the PAs were introduced to the teams. When PAs were first introduced to the location, there was strong opposition from some doctors who instead had preferred to take on more medical students.

Nobody has ever explained to me why, genuinely why they [PAs] are necessary other than sort of ‘we're hoping to plug some gaps on the cheap’. [Consultant ID:1, hospital 1]

In addition, the variability of the PA work led some staff, particularly in hospital 1, to ask for more clarity around the PA role within the team so that they can contribute to care more effectively.

They [PAs] often end up doing a similar job to a junior doctor and that is perhaps not the best use of their training. I think departments needs to re‐think how best PA's can be utilised to benefit the team and the PA's themselves. [Mid‐grade doctor ID:3, hospital 1]

Once employed the experience of integration varied, with some PAs given time to familiarise themselves with their environment, while others were expected to begin clinical work immediately.

Comparing analyses across the three hospitals revealed several differences at hospital 1 compared with hospitals 2 and 3. PAs and staff in hospital 1 presented the most negative and mixed attitudes, (Tables 3 and 4), which was supported by both open‐ended questions from questionnaire and interview statements. For example, as most PAs worked core hours only during the week, this caused some friction amongst staff working shifts and weekends. Some felt that advanced nurse practitioners were better qualified and cheaper than PAs and that they took away valuable training opportunities from junior doctors.

They [PAs] take away training opportunities from core trainees. We have nurse practitioners who are significantly better qualified and experienced than the PAs and are also cheaper. [Mid‐grade doctor ID:20, hospital 1]

Some PAs, particularly those based in hospital 1, were aware of negative attitudes towards their profession, but nevertheless tried to be philosophical about this.

Not everyone is entirely happy with the PA role and there are some.

differing opinions…but it is just a matter of containing and just being professional. [PA ID:1, hospital 1]

Some PAs who had noticed negative attitudes and lack of role clarity decided to address it themselves by becoming protagonists to raise their profile in the location and had seen some positive changes in how their role was perceived since their arrival in 2018.

Initially I had to educate both staff and patients about the role. However, it has been very rewarding to see others trust develop and progress the role. [PA ID:7, hospital 3]

In relation to how PAs compared themselves to others during member validation, a PA suggested that evidence of CPD could be provided with a portfolio on clinical practice, like other health professionals have and that is overseen be their clinical supervisor. All PAs said that they were able to gain the 50 CPD points needed for PAMVR. However, they also acknowledged that budget and time constraints limited other training opportunities, (e.g. endoscopy training), and attendance of external events and conferences. Five mid‐grade clinical staff and one consultant (hospital 1) felt that employment of PAs reduced training and surgical opportunities for doctors. In contrast, experienced PAs were keen to help train other clinicians, and staff noted that medical students often asked PAs to show them how to accomplish common procedures not included on their training syllabus.

Even though PAs cannot prescribe, PA participants felt that having pharmaceutical training would help them and the wider multiprofessional team with one of their common duties—clerking patients. Indeed, one suggested that healthcare professionals and students should learn together more.

It would be beneficial for PAs to be involved in group teaching, but to also attend teaching involving other healthcare professionals. [PA ID:7, hospital 3]

In hospitals 2 and 3, PAs were encouraged to join CPD provided for junior doctors where possible, but this was not the case at hospital 1.

It came out that the Junior Doctors did not want to have the Physician Associates being trained with them, which is really unfortunate. [Consultant ID:5, hospital 1]

As mentioned previously, only 64% of staff thought that PAs were adequately supervised in clinical work and felt that this may be a barrier to their employment (Table 4). When it came to the more holistic support provided during appraisals, findings highlighted clear differences between the hospitals. PAs in hospitals 2 and 3 felt they had good formal appraisals, but this did not happen for participating PAs in hospital 1.

I haven't had an appraisal, no one has actually sat with us and kind of decided where things are going and that is quite scary and then also there is no feedback, and because of that there is no feedback I don't really know how I am doing. [PA ID:1, hospital 1]

PAs who did not have appraisals felt isolated, did not have contact with other PAs, and seemed unaware of support available to them. By contrast, PAs working in hospitals 3 and 2 were also offered mentorship to help guide them through general matters, such as signing up for training. These hospitals (2 and 3) had recently appointed a senior member of staff to ensure PAs were properly managed and trained.

[The consultant] sorts out the appraisals and we have met a few times throughout the year to make sure that I am achieving the things that I want to achieve. [PA ID:1 hospital 2]

During member validation, PAs in hospitals 2 and 3 said that there were monthly meetings and social media support. In addition, there were opportunities to connect with PA ambassadors who were introduced by Health Education England to help raise awareness of the PA role and to liaise with education, sustainability and transformation teams.

Apart from gaining regulation, with the associated benefits including an enhanced clarity of the PA role, staff also noted the trade‐off between working regular hours versus career progression. Some staff members felt that the lack of career progression may reduce attraction of the PA role and others were critical of the development of PAs in the NHS.

I'm sure we are going to end up with a two‐tier system where you know, if you can pay you can see a doctor and if you can't, you will probably see a PA you know. [Consultant ID:1, hospital 1]

Findings suggest that hospitals 2 and 3 work to a strategic plan for employment of PAs in and across departments with staff shortages, whereas the hospital 1 functions more at departmental level.

[PA] posts in the hospital are not being funded by, at senior level so they have to be employed on the basis of individual departments, which obviously makes it a lot more challenging trying to get them into the workplace. [Consultant ID:5, hospital 1]

PAs in all hospitals noted that the hospital systems were not set up to differentiate what PAs could or could not order (e.g. ultrasound‐non‐ionising vs. X‐ray‐ionising).

Regardless of varied practices across the three hospitals, findings showed that PAs felt positive about their role. All PAs felt they were valued and trusted team members. Overall, PAs presented positive attitudes about the variety of work they did and enjoyed being part of the team.

I think we are all part of a big team and I think everyone appreciates that which is nice. [PA ID:1, hospital 2]

## 4 DISCUSSION

This study contributes to existing evidence showing the key contribution to Physician Associates (PAs) to the multiprofessional team and patient care. The study shows that the integration of PAs into hospital teams is variable and that there is a general lack of knowledge about the PA role amongst staff and patients. PAs in this study report that they are largely satisfied in their role but that they would welcome further support. Most staff embrace PAs as part of the team, but negative attitudes to this relatively new profession exist, something that the PAs are aware of, which highlights the need for a wider systems approach to support integration of PAs and more widely recognition of their valued contribution to patient care.

Indeed, patients in this study are predominately satisfied with the care they received from the PAs and state that it is especially due to PAs communicating appropriately and providing patient‐centred continuous care, which has been shown by others to be key to patients' experience (Drennan et al., 2019; Hooker et al., 2019; Taylor et al., 2019). The negative findings amongst staff revealed in hospital 1, may be a result of PAs not having as clearly specified roles in this hospital. This is a reminder about the importance of clarity around integration of roles (Roberts et al., 2019) and interprofessional practice.

A recent review found that interprofessional practice improved quality of care, but factors such as teamwork, leadership, organisational structure, communications and culture, moderate its effectiveness (Pomare et al., 2020). This study picks up on negative attitudes from some staff participants towards the PA role. Whether PAs are seen by those participants as complementing the multiprofessional team skillset, or in competition for training, may have influenced their attitudes. Drennan et al. (2019) noted that staff fully accepted PAs once they had shown their clinical capability.

PAs in this study are considered effective and efficient team members by many, though some clinicians question whether they can work safely, within their role. In addition, our research suggests that PAs add value to teams by teaching general clinical procedures to junior staff. Integration and acceptance of PAs in these hospitals is largely good. However, formal induction arrangements vary across hospitals, which is echoed in work presented by Drennan et al. (2019) where the PA employment experience differed in hospitals according to the strategic approach of using a clinical, or executive directorate, and funding streams.

Findings of this study show examples of where consultants and PAs identified gaps in the workforce, and leaders creatively helped fill such gap by recruiting PAs, highlighting good collaboration. However, findings also reveal confusion around management and formal supervision of PAs. Some PAs in this study do not feel supported or received appraisals, despite senior staff commitment to their employment. Hence, there appears to be a dissonance between the layers of leadership and/or management structure within at least one of the hospitals, where the necessary follow‐up is needed, to ensure sufficient and appropriate training and support is provided. Differences in training opportunities observed across the three hospitals suggest that in hospital 1 medical students and junior doctors influence who can join in‐house training sessions and did not want to include PAs. Reasons for their apparent reluctance to include PAs might be due to numerous factors, such as negative attitudes towards this new role. Many staff and PAs in this study emphasise the importance of continuity of care provided by PAs and how they improve the patient experience as valued members of the multiprofessional team.

Understanding the importance of clarifying roles and promoting positive attitudes towards different professions within the multiprofessional team can be achieved by offering purposeful interprofessional learning (IPL) opportunities (Hawkes et al., 2013). IPL can emphasise the need for teamwork, collaboration, co‐ordination and networking in the curricula, from the beginning of healthcare professionals' education and throughout their careers the practice setting, to enhance interprofessional collaborative practice and good patient care (Eddy et al., 2016; Hawkes et al., 2013; Xyrichis et al., 2018). In the context of this study, such learning opportunities may help raise the profile of PAs and their transition into the workplace at an early level. This is particularly important when introducing a new profession such as PA, not only to clarify the PA role but also others where roles may overlap to some extent as results show in this study. Here, we see a need for several staff to articulate, and in cases adapt their role, so that everyone is clear on their contribution to patient care and recognised as valued members of the multiprofessional team working interprofessionally.

Considering the findings from this study, we recommend that all hospitals carefully and strategically plan for how they recruit PAs, prepare staff and teams accordingly prior to their arrival. That way, PAs can successfully be welcomed and integrated into existing teams and together they can all adapt and work together towards their common goal, which is to deliver high quality care as an interprofessional team.

Once PAs have arrived, staff should have equal access to relevant training and IPL opportunities with relevant staff to ensure best practice and patient care. Indeed, additional training, such as pharmacological skills, identified by PAs in this research, would be ideal to enhance interprofessional practice through IPL. Findings from this study also suggest that all PAs should have regular formal appraisals in addition to their daily clinical supervision, to ensure career development. Providing such strategic and supportive culture and practices is likely to overcome some of the issues reported in this study—especially those linked to negative attitudes and lack of trust. Active mentoring and/or coaching would further support the integration of this new role into teams. Importantly, hospitals and the wider system need to show joined‐up commitment to this long‐term investment of PAs as valued members of the workforce.

Further research linked to practising PAs will help understand how PAs and other new professions can be integrated as part of multiprofessional teams in the most successful way. Further studies are needed to identify what type of support PAs and teams find meaningful.

Looking at the strengths of this study, the mixed methods approach provided opportunities to triangulate findings and assess contextual differences between hospitals. The bottom‐up approach ensured discovery of new findings, which is important when there is a scarcity of research (Crouch & McKenzie, 2006). While validation by participants and practicing PAs who were not part of the study provided trustworthiness to the findings. Taking a pragmatic realist approach, provided participant perceptions of the work system from a range of settings, thus capturing a broad range of viewpoints.

### 4.1 Limitations

Having senior clinical collaborators in charge of recruitment may explain the low number of participants. For example, the lack of responses from patients treated at hospital 3, may be due to ward staff not having sufficient time to approach staff and patients to ask if they were able to participate. Other limitations for the study include low numbers of PAs working in the location. Also, those participating were mostly female, which may have influenced the findings and thus the generalisability to a sample that were more gender balanced.

## 5 CONCLUSION

This study provides a pragmatic realist insight into PA contribution to patient care in a location where they have worked since 2018. Findings indicate that this relatively new profession could help address the global shortage of medical and nursing workforce. With that in mind, and taking findings of this study into account, it is important that hospitals and educational providers help support the integration of PAs. By clarifying the PA role to staff and patients, supporting teams to work together and investing in training opportunities for all is likely to benefit everyone. PAs will require formal and informal support, particularly during the initial transition into existing teams and the hospital itself, but this study has emphasised the need of the whole team to be supported for this integration to be successful. The study shows that a pro‐active approach by senior leaders and managers can help shape a culture where everyone feels valued and thus prevent development of negative attitudes. In order to further promote positive attitudes towards PAs and increase understanding of roles, opportunities for interprofessional learning throughout education and training can further improve how team members best work together to provide optimal healthcare for people as part of a system that is in much need for the kind of support PAs can offer.

## AUTHOR CONTRIBUTIONS

RB and SL designed the study. SHW collected all the data. SHW and SL, analysed and interpreted the data, and drafted the manuscript with the contribution of AG and JR. All authors have revised the manuscript critically and approved the final version.

## FUNDING INFORMATION

This research received no specific grant from any funding agency in the public, commercial or not‐for‐profit sectors.

## CONFLICT OF INTEREST STATEMENT

The authors have no competing interests to declare.

## ETHICS STATEMENT

Ethical approval was gained from the NHS, using the Integrated Research Application System, application number 263045. Approval was granted on condition the a senior clinical collaborator in each hospital would recruit participants.

## Data Availability

Data available on request due to privacy/ethical restrictions.

## References

[nop21655-bib-0001] Braun, V. , & Clarke, V. (2006). Using thematic analysis in psychology. Qualitative Research in Psychology, 3, 77–101.

[nop21655-bib-0002] Creswell, J. W. A. P. C. V. L. (2011). Designing and conducting mixed methods research (2nd ed.). Sage Publications.

[nop21655-bib-0003] Crouch, M. , & McKenzie, H. (2006). The logic of small samples in interview‐based qualitative research. Social Science Information, 45(4), 483–499.

[nop21655-bib-0004] Drennan, V. M. , Halter, M. , Wheeler, C. , Nice, L. , Brearley, S. , Ennis, J. , Gabe, J. , Gage, H. , Levenson, R. , de Lusignan, S. , Begg, P. , & Parle, J. (2019). What is the contribution of physician associates in hospital care in England? A mixed methods, multiple case study. BMJ Open, 9(1), e027012. 10.1136/bmjopen-2018-027012 PMC6359738 30700491

[nop21655-bib-0005] Eddy, K. , Jordan, Z. , & Stephenson, M. (2016). Health professionals' experience of teamwork education in acute hospital settings: A systematic review of qualitative literature. JBI Database of Systematic Reviews and Implementation Reports, 14(4), 96–137. 10.11124/jbisrir-2016-1843 27532314

[nop21655-bib-0006] Faculty of Physician Associates . (2021). Continuiing professional development.

[nop21655-bib-0007] Halter, M. , Wheeler, C. , Pelone, F. , Gage, H. , de Lusignan, S. , Parle, J. , Grant, R. , Gabe, J. , Nice, L. , & Drennan, V. M. (2018). Contribution of physician assistants/associates to secondary care: A systematic review. BMJ Open, 8(6), e019573. 10.1136/bmjopen-2017-019573 PMC6020983 29921680

[nop21655-bib-0008] Hawkes, G. , Nunney, I. , & Lindqvist, S. (2013). Caring for attitudes as a means of caring for patients‐‐improving medical, pharmacy and nursing students' attitudes to each other's professions by engaging them in interprofessional learning. Medical Teacher, 35(7), e1302–e1308. 10.3109/0142159x.2013.770129 23581855

[nop21655-bib-0009] Hooker, R. S. , Moloney‐Johns, A. J. , & McFarland, M. M. (2019). Patient satisfaction with physician assistant/associate care: An international scoping review. Human Resources for Health, 17, 104. 10.1186/s12960-019-0428-7 31881896 PMC6935095

[nop21655-bib-0010] Mittman, D. E. , Cawley, J. F. , & Fenn, W. H. (2002). Physician assistants in the United States. BMJ, 325(7362), 485–487. 10.1136/bmj.325.7362.485 12202333 PMC1124003

[nop21655-bib-0011] Pomare, C. , Long, J. C. , Churruca, K. , Ellis, L. A. , & Braithwaite, J. (2020). Interprofessional collaboration in hospitals: A critical, broad‐based review of the literature. Journal of Interprofessional Care, 34(4), 509–519. 10.1080/13561820.2019.1702515 31928245

[nop21655-bib-0012] Rick, T. J. , & Ballweg, R. (2017). Physician assistants and the expanding Global Health‐care workforce. The American Journal of Tropical Medicine and Hygiene, 97(3), 643–644. 10.4269/ajtmh.17-0176 28722636 PMC5590609

[nop21655-bib-0013] Ritsema, T . (2018). Faculty of physician associates census results 2018. Royal College of Physicians. https://fparcp.co.uk/about‐fpa/fpa‐census

[nop21655-bib-0014] Roberts, S. , Howarth, S. , Millott, H. , & Stroud, L. (2019). What can you do then? Integrating new roles into healthcare teams: regional experience with physician associates. Future Healthcare Journal, 6(1), 61–66. 10.7861/futurehosp.6-1-61 PMC6520076 31098589

[nop21655-bib-0100] Robson, C. (2002). Real world research (2nd ed.). Blackwell.

[nop21655-bib-0015] Taylor, F. , Halter, M. , & Drennan, V. (2019). Understanding patients' satisfaction with physician assistant/associate encounters through communication experiences: A qualitative study in acute hospitals in England. Health Services Research, 19, 603. 10.1186/s12913-019-4410-9 31455342 PMC6712610

[nop21655-bib-0016] Timmermans, M. J. C. , van Vught, A. J. A. H. , Van den Berg, M. , Ponfoort, E. D. , Riemens, F. , Van Unen, J. , Wobbes, T. , Wensing, M. , & Laurant, M. G. H. (2016). Physician assistants in medical ward care: A descriptive study of the situation in The Netherlands. Journal of Evaluation in Clinical Practice, 22(3), 395–402. 10.1111/jep.12499 26695837

[nop21655-bib-0017] WHO . (2016). High level commission on health employment and economic growth. https://www.ilo.org/global/about‐the‐ilo/mission‐and‐objectives/lang‐‐en/index.htm 10.12927/whp.2017.25309 29400270

[nop21655-bib-0018] Xyrichis, A. , Reeves, S. , & Zwarenstein, M. (2018). Examining the nature of interprofessional practice: An initial framework validation and creation of the InterProfessional activity classification tool (InterPACT). Journal of Interprofessional Care, 32(4), 416–425. 10.1080/13561820.2017.1408576 29236560

[nop21655-bib-0019] Yin, R. K. (2009). Case study research design and methods. Sage Publications.

